# Genetically Engineered Vaccinia Viruses As Agents for Cancer Treatment, Imaging, and Transgene Delivery

**DOI:** 10.3389/fonc.2017.00096

**Published:** 2017-05-23

**Authors:** Dana Haddad

**Affiliations:** ^1^Department of Radiology, Memorial Sloan-Kettering Cancer Center, New York, NY, USA

**Keywords:** oncolytic viral therapy, vaccinia virus, molecular imaging, gene therapy

## Abstract

Despite advances in technology, the formidable challenge of treating cancer, especially if advanced, still remains with no significant improvement in survival rates, even with the most common forms of cancer. Oncolytic viral therapies have shown great promise for the treatment of various cancers, with the possible advantages of stronger treatment efficacy compared to conventional therapy due to higher tumor selectivity, and less toxicity. They are able to preferentially and selectively propagate in cancer cells, consequently destroying tumor tissue mainly *via* cell lysis, while leaving non-cancerous tissues unharmed. Several wild-type and genetically engineered vaccinia virus (VACV) strains have been tested in both preclinical and clinical trials with promising results. Greater understanding and advancements in molecular biology have enabled the generation of genetically engineered oncolytic viruses for safer and more efficacious treatment, including arming VACVs with cytokines and immunostimulatory molecules, anti-angiogenic agents, and enzyme prodrug therapy, in addition to combining VACVs with conventional external and systemic radiotherapy, chemotherapy, immunotherapy, and other virus strains. Furthermore, novel oncolytic vaccinia virus strains have been generated that express reporter genes for the tracking and imaging of viral therapy and monitoring of therapeutic response. Further study is needed to unlock VACVs’ full potential as part of the future of cancer therapy.

## Introduction

Replication-competent oncolytic viral therapies have shown great promise preclinically and in clinical trials for the treatment of various cancers. They are able to preferentially and selectively propagate in cancer cells, consequently destroying tumor tissue mainly *via* cell lysis, while leaving non-cancerous tissues unharmed (1). Oncolytic vaccinia virus (VACV) strains have been of particular interest due to several advantages, including large genomic capacity, fast and efficient replication, and impressive safety profile (2, 3). In this review, an overview of replication-competent oncolytic vaccinia viruses is presented, with particular focus on its potential for cancer treatment, imaging, and transgene delivery.

## Why Vaccinia for Oncolytic Viral Therapy

There are several advantages of using vaccinia virus as an agent for oncolytic viral therapy. VACVs’ large 192-kb genome enables a large amount of foreign DNA to be incorporated without significantly reducing the replication efficiency of the virus, facilitating genetic engineering for safer attenuated viruses and transgene delivery (2). Cytoplasmic replication of the virus lessens the chance of recombination or integration of viral DNA into normal cells, and its DNA-based genome also makes it more stable than RNA-based viruses. It has been shown to be capable of evading the immune system and of infecting a wide variety of cells, enabling more effective systemic delivery. Perhaps most importantly, VACVs’ safety profile after its use as a live vaccine in the World Health Organization’s smallpox vaccination program in more than 200 million people makes it particularly attractive as an oncolytic agent and gene vector (3). Furthermore, vaccinia immunoglobulin and antiviral drugs are available if needed (4).

Vaccinia virus has a natural selectivity to tumors, with suggestion that leaky vasculature found in tumors being one of the major determinants of tropism (5, 6). It has also been shown that oncolytic viruses target cancers that overexpress proteins such as ribonucleotide reductase, DNA repair enzymes, and anti-apoptotic proteins; characteristics that tend to make tumor cells resistant to chemotherapy and radiation therapy (7, 8). Further selectivity of VACV has been achieved though the deletion of the thymidine kinase (TK) gene, involved in nucleotide synthesis, limiting viral replication to nucleotide rich cancer cells (6, 9–11). More investigation is needed to elucidate the exact mechanisms rendering vaccinia viruses highly selective and oncogenic in tumors, with more recent studies also utilizing microarray analysis and pathway analysis for further understanding (12).

In addition to their oncotropic and oncolytic effects, replication-selective vaccinia viruses can be used for transgene delivery to facilitate imaging of viral replication and enhance the probability of tumor eradication through multiple avenues (13). Replication-selective viral systems can employ endogenous viral gene expression control signals (promoter/enhancer, polyadenylation, and splice signals) for transgene expression. The use of endogenous viral promoters may also allow more predictable and controlled transgene expression (14).

## History of Vaccinia Viruses as Oncolytic Virotherapies

Levatidi’s laboratory was the first to discover that VACVs were naturally oncolytic (15). Cassel and Garrett followed this by successfully treating murine malignant ascites (16). In a case report by M.D. Anderson, inadvertent administration of the vaccinia virus resulted in remission of chronic lymphocytic leukemia (CLL) in a patient (17). Another patient had remission of his CLL for more than 3 years, although becoming ill from his vaccinia vaccination and was successfully treated with immunoglobulin therapy (18). A different patient with multiple myeloma had a partial response after intravenous administration of vaccinia virus (19). Partial remissions have also been reported in patients with metastatic renal or pulmonary carcinomas (19, 20). These findings lead to several clinical trials including treating melanoma with a potent vaccinia virus encoding granulocyte-macrophage colony-stimulating factor (GM-CSF) (21, 22). Ongoing clinical trials are discussed in the following sections in this review.

While early studies and trials were considered ground breaking, interest in viruses as anti-neoplastic therapies was abandoned due to unimpressive and short-lived success, as well as unacceptable side effects that ended some trials (23). It is only in the past 3 decades that the fervor of viruses as a strategy against cancer has been reignited with the advancement in scientific knowledge and technology. We now possess tools that enable us to develop more targeted and effective viruses (24).

## Development of Newer VACV Generations

Due to the advantages of vaccinia viruses, several preclinical trials have been performed in a variety of cancer origins. The use of oncolytic vaccinia viruses derived from several strains, including WR, LIVP, Wyeth, Copenhagen, revealed that WR-derived strains were able to colonize tumors in human xenografts in nude as well as syngeneic tumors in immunocompetent wild-type animals (25, 26).

Greater understanding and advancements in molecular biology have enabled development of a generation of genetically engineered oncolytic viruses for safer and more efficacious treatment. One of the earliest examples of the development and use of a recombinant VACV was given by Timiryasova and colleagues, who investigated the use of VACV and recombinant derivatives, recVV2, rVV-p53, on the growth of C6 rat glioma cells in an athymic nude mice model. They found that VACV effectively infected C6 cells *in vitro*, inducing high level of foreign gene expression, including rW-p53-mediated expression of the tumor suppressor p53 protein. In C6-implanted nude mice, injection of VACV or rVV-p53 induced effective inhibition of tumor growth in comparison to control groups, with a greater effect with rVV-p53, apparently due to overexpressed p53 and p53-mediated cell apoptosis. These results, and others, paved the way for the use of vaccinia-mediated delivery of therapeutic genes represent novel potential strategies for tumor therapy (27). Since then, the successful use of vaccinia virus as an oncolytic agent has been so far published in at least 50 human tumor models (Table 1). Moreover, systemic treatment with vaccinia virus was shown to reduce metastatic burden, demonstrated with an aggressive PC-3 prostate cancer model (28) and in rabbits bearing VX2 liver tumors (29, 30).

**Table 1:** **Vaccinia virotherapy in preclinical human tumor models**.

Vaccinia virus	Tumor type	Tumor model	Reference
LIVP	Prostate	PC-3	(31, 32)
		DU-145	(30–32)
	Pancreatic	Mia-Paca2	(30, 32–34)
		PANC-1	(32, 33, 35)
		Suit-2	(36)
	Breast	GI-101A	(32, 37)
	Lung	A549	(30, 32)
	SCC	MSKQLL2	(38)
	Mesothelioma	MSTO-211H	(39)
	Thyroid	8505C	(40)
		DRO90-1	(40)
	Ovarian	OVCAR-3	(41)
		ES2	(42)
	Melanoma	1858-MEL	(41)
		888-MEL	(41)

WR	Renal	786-O	(43)
		ACHN	(43)
		769P	(43)
		Renca	(43)
	Multiple myeloma	My5	(44)
		RPMI8226	(44)
	Colorectal	HCT116	(29, 45)
	Ovarian	HT29	(46)
		UCI-101	(47)
		SKOV-3	(47)
		A2780	(48, 49)

WR (vvDD)	Brain	U87MG	(50)
		U118	(50)

Copenhagen	Colorectal	LoVo	(51)

**Figure 1:**
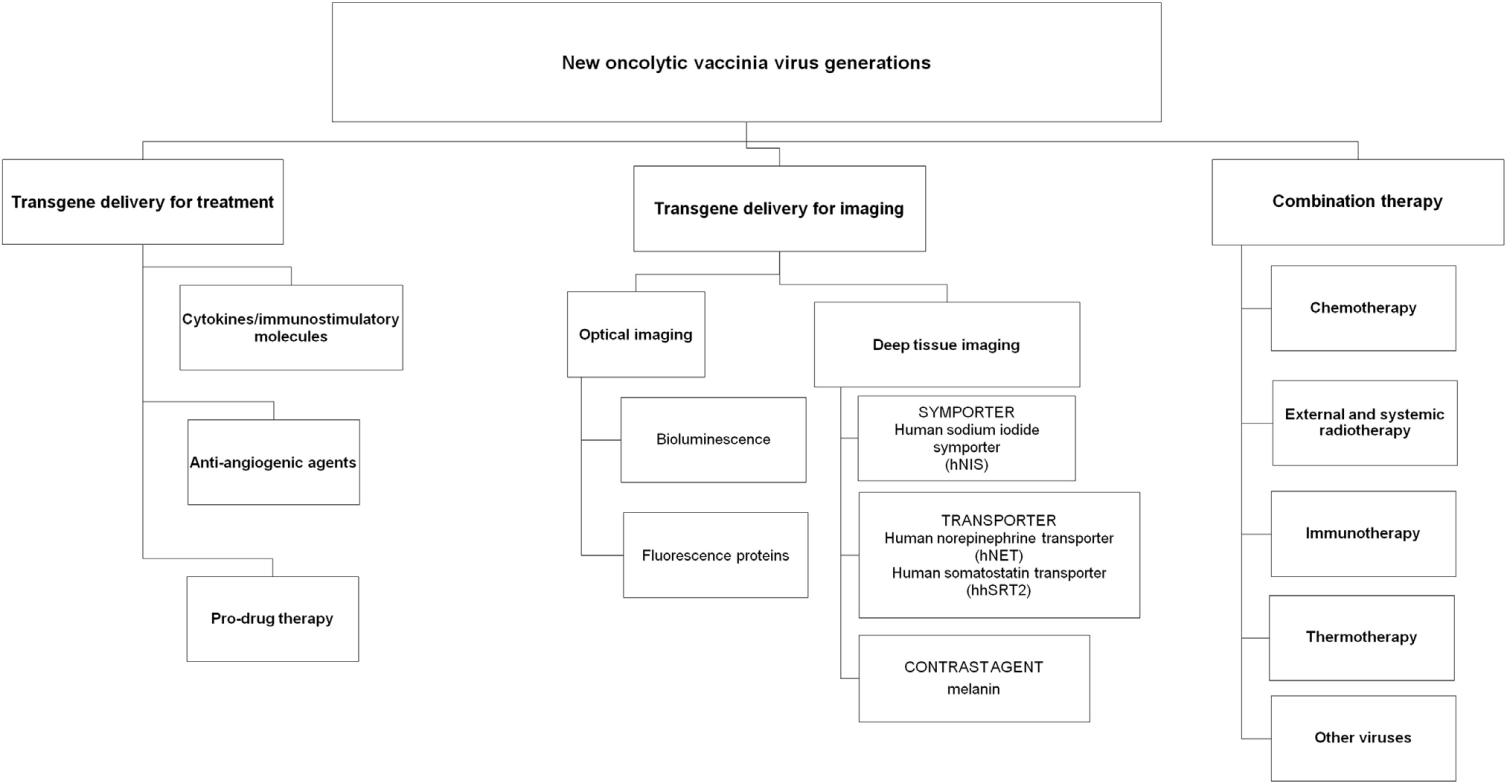
**Overview of new oncolytic vaccinia virus generations**.

Several strategies have been investigated with vaccinia viruses for the treatment of human cancers, including arming VACVs with immunostimulatory molecules and anti-angiogenic agents, utilizing VACVs as delivery agents for targeted enzyme prodrug therapy and reporter gene expression for imaging and combining VACVs with other cancer treatments including immune-, chemo- and radiotherapy (Figure 1).

### VACVs Armed with Cytokines/Immunostimulatory Molecules

Combining viral therapy with cytokines has been attempted with the aim of harnessing the host’s own immunity to assist tumor rejection and destruction. One of the earliest examples of this was the development of vaccinia virus strains encoding human GM-CSF, JX-549, and JX-963, which were shown to enhance antitumor immunity due to the expression of the GM-CSF transgene *in situ* (46, 52). Direct oncolysis plus GM-CSF expression stimulated the shutdown of tumor vasculature and antitumoral immunity, significantly reducing tumor burden and increasing median survival. Tumor-specific virus replication and gene expression, systemically detectable levels of GM-CSF, and tumor-infiltrating cytotoxic T-cells (CTLs) as well as significant increases in neutrophil, monocyte, and basophil concentrations in the peripheral blood were also demonstrated.

Vaccinia expressing co-stimulatory cytokines have even also been shown to help overcome the tumor microenvironment’s immune suppressive characteristics. The melanoma microenvironment in particular leads to local T-cell tolerance in part through down-regulation of co-stimulatory molecules, such as B7.1 (CD80). A 2-dose-escalation phase I clinical trial was conducted with 12 patients using a recombinant vaccinia virus expressing B7.1 for monthly IT vaccination of accessible melanoma lesions. The approach was well tolerated with only low-grade fever, myalgias, and fatigue reported, with two patients experiencing vitiligo (28). An objective partial response was observed in one patient and disease stabilization in two patients, one of whom was alive without disease 59 months following vaccination. All patients demonstrated an increase in post-vaccination antibody and T-cell responses against vaccinia virus.

Cytokines have also been utilized to increase the tumor selectivity. Kirn et al. developed a vaccinia virus strain expressing the cytokine IFN-β, JX-795, which is incapable of responding to this cytokine to have the dual benefits as a cancer therapeutic with increased anticancer effects and enhanced virus inactivation in normal tissues (29). The virus was based on a vaccinia B18R deletion mutant backbone for IFN-β expression, as the B18R gene product neutralizes secreted type-I IFNs. JX-795 had superior tumor selectivity and systemic efficacy when compared to the TK-/B18R- control or wild-type vaccinia in preclinical models. The authors concluded that by combining IFN-dependent cancer selectivity with IFN-β expression to optimize both anticancer effects and normal tissue antiviral effects, tumor-specific VIRAL replication, IFN-β gene expression, and treatment efficacy were achieved following systemic delivery in preclinical models.

### VACV Delivering Anti-Angiogenic Agents

Further improvement of oncolytic potential was studied by attempting to inhibit tumor vasculature *via* expression of an endostatin/angiostatin fusion gene, targeting the vasculature endothelial growth factor (VEGF) (30, 36, 43). VEGF binds to specific receptors on epithelial cells and is a major player in tumor angiogenesis. Inhibition of VEGF has been extensively studied in several cancer models (43, 53–56), with Avastin being one of the most successful immunotherapeutic proteins to date. This drug has been approved by the US Food and Drug Administration (FDA) for use in combination with chemotherapy for the treatment of metastatic colorectal cancer and most forms of metastatic non-small cell lung cancer (57, 58). Vaccinia-mediated blocking of VEGF was achieved by either fusing the VEGF receptor 1 to the Fc tail of human IgG antibody (VEGFR-1-Ig) or secretion of a single-chain antibody (GLAF-1) to VEGF. In both cases, VEGF was bound and thus prevented interaction to its natural receptors on endothelial cells resulting in lower blood vessel densities within the tumor tissue. The reduced tumor vascularity was accompanied by faster regression of tumors; although in one study, this depended on the dose of virus injected (43). In the same study, the VEGFR-1-Ig encoding vaccinia virus strain was found to be more lethal to mice than the parental strain. For the GLAF-1 encoding virus strains, no changes in toxicity were described.

### Use of VACVs in Gene-Directed Enzyme Prodrug Therapy

Another approach to enhance the oncolytic effects caused by vaccinia virus strains is the so-called gene-directed enzyme prodrug therapy (GDEPT). In this article, a relatively non-toxic prodrug is enzymatically converted to toxic drugs which result in killing of the enzyme-producing tumor cells. Moreover, the so-called bystander effect caused by diffusion of the drug into neighboring cells results in killing of cells in close proximity to the enzyme-producing cell even if they were not made to express the prodrug converting protein.

The most prominent enzyme type in vaccinia virus-mediated GDEPT is cytosine deaminase, which is absent in mammalian cells and used in combination with 5-fluorocytosine (10, 48, 51, 59, 60). This prodrug is converted to 5-fluorouracil, whereby the efficiencies depend on the specific cytosine deaminase (e.g., bacterial and fungal) and the presence of uracil phosphoribosyltransferase (61, 62). When using this system in combination with oncolytic vaccinia virus strains, the reported results indicate better therapeutic effects when compared to the oncolytic virus alone. However, the therapeutic benefit was expected to be higher (48). In other studies, similar results were found when using a β-galactosidase-expressing vaccinia virus strain in combination with an inducible prodrug seco-analog of duocarmycin SA (63). Several reasons might be responsible for these observations: first, the rapid kinetics of oncolytic vaccinia virus replication might functionally overlap with the used prodrug system; and second, the administration of prodrug may have inhibited the viral replication, thus reducing the antitumoral cytotoxicity induced by the oncolytic virus itself. This effect has already been reported by McCart et al. (64) but was not observed in all prodrug systems (63). Different dosing schemes or other GDEPT systems should still be considered and might cause stronger synergistic effects between the oncolytic virus strain and prodrug therapy.

## Vaccinia Viruses for Cancer Imaging

Oncolytic vaccinia virotherapy has shown success in preclinical trials and much promise in completed and ongoing human clinical trials. However, biopsy is the current gold standard for monitoring the therapeutic effects of viral oncolysis (65). This may be feasible in preclinical trials, or early clinical trials; however, a non-invasive test facilitating ongoing monitoring of therapy is needed for human studies (66). This would enable the assessment of the biodistribution of oncolytic viruses to ensure safety and correlation with treatment efficacy, as well as the potential for a more sensitive and specific diagnostic technique to detect tumor origin and, more importantly, the presence of metastases (67).

Consequently, novel oncolytic vaccinia virus strains have been generated that express reporter genes such as green fluorescent protein (*GFP*), *RLuc* for optical imaging, and the human somatostatin receptor type 2, the human norepinephrine transporter (*hNET*), and the human sodium iodide symporter (*hNIS*), which selectively bind radiotracers and therefore should also be detectable in deep tissues of humans (35, 59).

Several non-invasive imaging methods are already in clinical use, including optical methods using fluorescence and bioluminescence, as well as deep tissue imaging modalities utilizing instrumentation such as positron emission tomography (PET) and single photon emission computed tomography (SPECT).

### Optical Imaging

Optical detection methods such as fluorescence and bioluminescence have the advantage of short acquisition times (for fluorescence imaging, few milliseconds to several seconds, and for bioluminescence, a few seconds to several minutes), and high spatial resolution. The major disadvantage of optical imaging is the inability to perform deep tissue imaging due to autofluorescence, light scattering, and the opacity of tissues to light below 600 nm due to absorbance by hemoglobin. Nevertheless, optical imaging in small animals has been and still is a very important tool to follow the distribution of oncolytic vaccinia viruses equipped with genes for luciferases (25, 26, 36, 42, 46, 47, 68–70) or fluorescent proteins such as GFPs (26, 31, 33, 37, 39, 44, 47, 50, 68, 70). Moreover, a GFP encoding vaccinia virus strain, GLV-1h68, is currently in clinical phase I and II trials in which this fluorescent protein can be used to monitor the colonization of near-surface tumors and metastases (71). The discovery of new fluorescent proteins in the near-infrared spectrum will probably result in the ability to detect oncolytic viruses in somewhat deeper tissues (72).

### Deep Tissue Imaging

In contrast to optical imaging, deep tissue imaging modalities can be used for non-invasive deep tissue imaging utilizing radiotracers with differing properties. These radiotracer imaging technologies are able to measure the distribution of radiotracers in the human body (73). They are widely available and have a wide range of clinical and research applications. Two classes of clinical nuclear imaging systems exist: those designed to image single gamma-emitting radionuclides such as 99m-technetium pertechnetate (^99m^TcO_4_) and Iodine-131 (^131^I) and those designed to image positron-emitting radionuclides such as fluorine-18, carbon-11, and Iodine-124 (^124^I). The single gamma-emitting imaging system is referred to as single photon imaging or, when performed tomographically, single photon emission computed tomography (SPECT). The positron-emitting imaging system is known as PET. PET has greater spatial resolution and higher sensitivity and is easier to quantify than SPECT.

Viral gene expression during the lytic phase of the viral life cycle of vaccinia virus is highly regulated and can be broadly classified into three serially activated phases: immediate-early (IE), early (E), and late (L) (14). Based on the expression of endogenous viral genes, it may be possible to predict the expression kinetics (timing and expression levels) of the transgene(s) carried by the replicating agent. Furthermore, when multiple transgenes are inserted into a single virus, their expression may be orchestrated to occur simultaneously or serially, at levels that will maximize their therapeutic benefit. Expressing transgenes serially at different times in the viral lytic cycle is of greatest value early in treatment when the infection may be more synchronized. As a viral infection spreads and encounters a heterogeneous tumor cell mass, it will likely become asynchronous, although the relative expression of different transgenes may still be maintained.

#### Human Somatostatin Receptor 2 (SSTR2)

The SSTR2 is targeted by the high-affinity synthetic peptide pentetreotide, which is commonly used for receptor imaging after being radiolabeled with indium-111 (74). This receptor is expressed in normal human kidney cells and neuroendocrine tumors, and gene therapy approaches have also been attempted to deliver the SSTR2 to non-expressing tumors using adenoviral vectors (74, 75). In a study by McCart et al., nude mice bearing subcutaneous murine colon CA xenografts were injected intraperitoneally with an *SSTR2*-expressing VACV or control and imaged 6 days later with ^111^In-pentetreotide-mediated SPECT. Tumors infected with the SSTR2-expressing VACV accumulated higher concentrations of radioactivity compared to tumors in animals receiving the control virus. Further, SSTR2-infected tumors were visible on imaging 6 days after VACV injection and could be visualized for up to 3 weeks post viral injection using repeat radiotracer injections (59). Limitations of the SSRT2 receptor are that radiotracers for SSRT2 require prior radiolabeling for accumulation of radioprobes and the 1:1 binding relationship with radiolabeled limiting signal amplification.

#### Human Norepinephrine Transporter

Another deep tissue reporter gene investigated in oncolytic viral strains is the *hNET*. hNET is a cell surface human protein mediating the transport of norepinephrine, dopamine, and epinephrine across the cell membrane. It can be imaged by SPECT or PET using the radiotracer meta-iodobenzylguanidine (MIBG) (76, 77). The use of the hNET-MIBG reporter imaging is attractive since it is of human origin and will unlikely induce an immune response, as well as its limited expression in the central and peripheral sympathetic nervous systems (78). An oncolytic vaccinia virus carrying *hNET*, GLV-1h99 derived from GLV-1h68, mediated the expression of the hNET protein on the cell surface of infected tumor cells, resulting in specific uptake of the radiotracer [^131^I]-MIBG (35). In mice, GLV-1h99-infected pancreatic tumors were readily imaged by [^124^I]-MIBG-PET. This virus further mediated imaging of an orthotopic mouse model of human malignant mesothelioma using both ^123^I-MIBG-mediated SPECT imaging and ^124^I-MIBG-mediated PET imaging (79).

#### Human Sodium Iodide Symporter

The *hNIS* is an intrinsic plasma membrane protein which mediates the active transport and concentration of iodide in the thyroid gland cells and some extra thyroidal tissues, in particular, the lactating mammary gland, as well as in the stomach, salivary glands, skin, brain, spleen, small intestine, ovaries, prostate, and testes (80).

**Figure 2:**
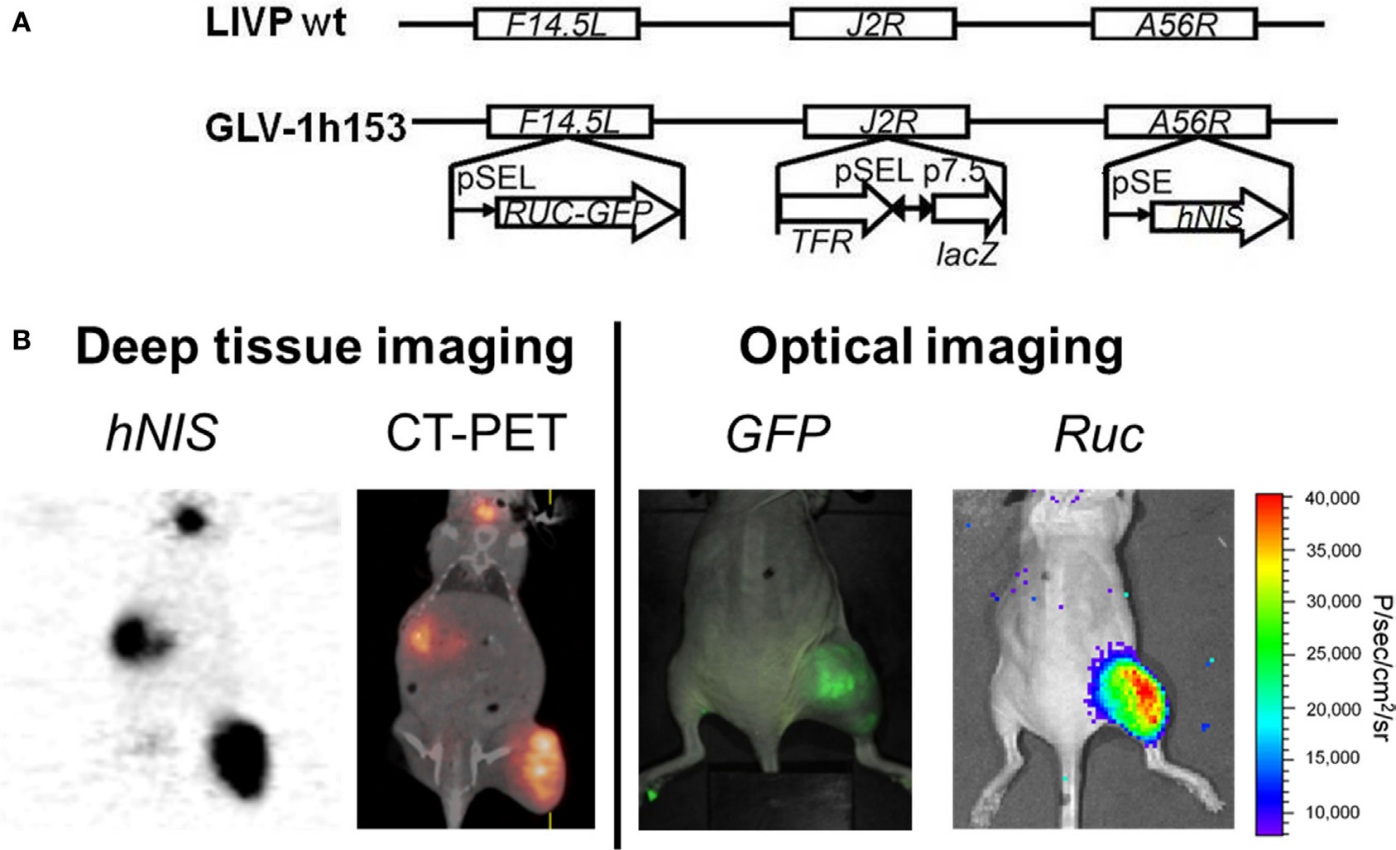
**Molecular imaging of oncolytic vaccinia virus GLV-1h153**. **(A)** GLV-1h153 construct. GLV-1h153 was derived from LIVP-wt virus, by replacing the gusA expression cassette at the A56R locus with the human sodium iodide symporter (hNIS) expression cassette through homologous recombination. The virus also contains RUC-green fluorescent protein (GFP) and lacZ expression cassettes at the F14.5L and J2R loci, respectively. PE, PE/L, P11, and P7.5 are the vaccinia virus synthetic early, synthetic early/late, 11K, and 7.5K promoters, respectively. TFR is a human transferrin receptor inserted in the reverse orientation with respect to the promoter PE/L. **(B)** GFP, bioluminescence, and hNIS signal could be detected in GLV-1h153-infected tumors. Fusion of PET and CT images correlated hNIS-mediated uptake signal anatomically to location of thyroid and stomach due to intrinsic hNIS expression, bladder due to radiotracer excretion, and tumor due to virus-mediated hNIS expression. Virally-mediated GFP and bioluminescence signals located only to tumor, demonstrating tumor-specific viral replication.

*hNIS* gene transfer *via* viral vector may allow infected tumor cells to concentrate several easily attainable, commercially available, and relatively inexpensive, carrier-free radioisotopes such as ^123^I, ^124^I, ^125^I, ^131^I, ^99m^TcO_4_, rhenium, and astatine for non-invasive imaging of *NIS* expression, all of which have long been approved for human use. The first vaccinia virus carrying the hNIS was GLV-1h153, also a derivative of GLV-1h68 (81). The virus also encoded for the *GFP* and the *RLuc* genes, and it was found to be successful in fluorescence, bioluminescent, and deep tissue image monitoring of viral replication and therapy (Figure 2) (82, 83). Moreover, GLV-1h153 successfully regressed several tumor types in preclinical models including pancreatic cancer, triple negative breast cancer, gastric cancer, malignant pleural mesothelioma, and most recently, prostate cancer (84–87).

#### Melanin

A recent study by Stritzker et al. explored the use of a VACV encoding for the production of a contrast agent, melanin (88). The oncolytic virus-mediated production of melanin and its optical absorption in the near-IR spectrum enabled the imaging of A549 tumors and metastases *via* the utilization of magnetic resonance imaging and multi-spectral tomography. The ubiquitous presence of melanin in all kingdoms of life suggests that the introduction of melanin synthesis as a diagnostic and theranostic marker is possible in most species.

## Combination Therapies with VACVS

Although the therapeutic effect of vaccinia virus shows promise, combining conventional therapies may enhance oncolytic viral treatment and help circumvent the immune system for optimal delivery of viruses to tumors.

### Chemotherapy

Combination of oncolytic vaccinia virus with classical chemotherapeutic agents such as gemcitabine and cisplatin led to accelerated tumor size reduction compared to monotherapy using VACV alone (89, 90). At the same time, each of the chemotherapeutics could only slow down tumor growth but did not result in complete tumor regression. For example, combination treatment with VACV GLV-1h68 and cyclophosphamide significantly improved the antitumor efficacy of GLV-1h68 and led to an increased viral distribution within the tumors (89). Pro-inflammatory cytokines and chemokines were distinctly elevated in tumors of GLV-1h68-treated mice. Factors expressed by endothelial cells or present in the blood were decreased after combination treatment. A complete loss in the hemorrhagic phenotype of the PC14PE6-RFP tumors and a decrease in the number of blood vessels after combination treatment could be observed.

In another study by Ottolino-Perry and colleagues, a VACV expressed the human somatostatin receptor and red fluorescent protein, vvDD-SR-RFP, with oxaliplatin or SN-38 (active metabolite of irinotecan) in colorectal cancer cell lines *in vitro* (91). Utilizing the Choui–Talalay method for determining drug–drug interactions, they were able to show that combination therapy induced additive and synergistic effects in different cell lines, which also depended on doses of treatment utilized. The VACV was then combined with irinotecan in an orthotopic model of metastatic colorectal cancer. Combination therapy was well tolerated in tumor-bearing mice, with a significant increase in the median survival compared to control groups, including either treatment alone. Increased apoptosis following combination therapy was also observed. Combination of oncolytic VACV with other chemotherapeutic agents in future studies will provide useful data as to which combination therapies are best suited for each type of cancer.

### Radiation Therapy

Radiation therapy has also been explored as a possible strategy against malignancies in combination with oncolytic viral therapy (90, 92, 93). Radiotherapy can either be local in the form of external beam radiation therapy (EBRT), or systemically administered. OVs may act as radiosensitizers by affecting pathways that render tumors resistant to treatment. Further, the selective cytotoxicity of viruses to tumors may enable more targeted radiotherapeutic strategies especially with systemically administered radiotherapies. In preclinical models, the combination of VACV and radiotherapy significantly delayed tumor growth and prolonged survival compared to single agent therapy in several cancers such as prostate and sarcoma (94–96), with data suggesting that virally mediated down-regulation of anti-apoptotic proteins may increase the sensitivity of tumor cells to the cytotoxic effects of ionizing radiation (96).

Vaccinia viruses encoding transporter genes such as *hNIS* have also been found to have a synergistic antitumor effect when combined with systemic ionizing radiation, such as ^131^I (85, 97). One mechanism for such synergy appears to be radiation-induced upregulation of certain cellular DNA repair genes that result in promoting viral replication (7, 98). Furthermore, a bystander effect may be possible as ^131^I undergoes alpha particle decay with a path length of 0.2–2.4 mm (99). If additive or synergistic effect is found, patients may be more safely treated with combinations of lower doses of virus and radioiodine. Application of carrier-free radioiodine would thus be extended, and the extensive experience with radioiodine in thyroid cancer management will undoubtedly be helpful in the treatment of other *NIS*-transfected tumors. Our laboratory demonstrated an enhanced effect of oncolytic viral therapy with GLV-1h153 when combined with radiotherapy ^131^I in both pancreatic and breast cancer xenografts (97).

### Immunotherapy

The mechanisms in which tumors ‘escape’ immune surveillance have long been a topic of much investigation. The immune surveillance theory, also referred to as “cancer immunoediting,” is typically characterized by three main phases: elimination; equilibrium; and eventually, escape (100). Tumors are believed to escape surveillance when the adaptive immune system fails to recognize tumor cells as foreign or dangerous to the host (101). Evidence in murine models has shown that tumors that do not enter the lymph nodes (or are compartmentalized from T-cells) failed to alert adaptive responses, and thus are ‘ignored’ by the immune system (102). However, CTL responses were induced by direct interaction between tumor cells and T-cells. Therefore, mechanisms thought to enable tumor cells to be ‘ignored’ are mainly through alterations in the antigen-processing and presentation pathway. In particular, dendritic cells (DCs) are believed to be major characters in this immune response (103). They are considered the most potent of antigen-presenting cells, with antitumor effects due to their ability to induce CTL responses. Several studies utilizing dendritic cell vaccines have been conducted to understand the triggers of activation and maturation, as well as functioning mechanisms; however, limited success was yielded in clinical trials (47, 104). This may be due to the inability of CTLs to efficiently traffic to and disseminate into the tumor, or a suppressive local environment leading to loss of their cytotoxic potential or conversion into regulatory T-cells (104).

This suppressive environment may be mediated by immune checkpoints (105, 106). Immune checkpoints refer to inhibitory pathways crucial for maintaining self-tolerance and modulating the duration and amplitude of physiological immune responses to minimize damage. It is now known that certain tumors exploit immune checkpoint pathways as a major mechanism of immune evasion, particularly against T-cells that are specific for tumor antigens. Many of these immune checkpoints are initiated by ligand-receptor interactions, such as cytotoxic T-lymphocyte-associated antigen 4 (CTLA4) (107), as well as proteins such as programmed cell death protein 1 (PD-1/PDL-1) (108), which can be readily blocked by antibodies or modulated by recombinant forms of ligands or receptors (105).

Virotherapy may have the ability to harness the benefits of the host immune response while inhibiting undesirable components. It is hypothesized that under certain conditions, a strong local host immune response at the site of infection within the tumor can support and enhance antitumor potential of the virus (109), so in addition to direct oncolysis, it may be possible to induce an immune response against the virus and subsequently against the tumor itself. This may even lead to systemic clearance of tumor metastases expressing specific antigens.

Several groups have looked into the combination of VACVs with several forms of immunotherapy, including cancer vaccines and immune checkpoint blockade (104). Strategies to date include the combination of DC vaccination with oncolytic viruses expressing chemokines known to attract the T-cells produced into the tumor (110), or the combination of chimeric antigen receptor T-cells with oncolytic virus strains expressing both chemokines and cytokines to attract both these cells into the tumor and subsequently maintain their phenotype (111).

Furthermore, since CTLA4 antibodies were approved by the FDA, studies have also explored the potential of combining vaccinia virus with immune checkpoint blockade (105, 112). For example, one group showed that combination therapy with oncolytic vaccinia virus and anti-CTLA4 can effectively treat several cancer types (106). However, the benefits of combination therapy were dependent on the viral strains, in addition to timing of the treatment. Administering both treatments simultaneously resulted in loss of therapeutic benefit, probably due to early induction of anti-viral immunity, dampening the effects of oncolysis. When the antibody was administered 3 days post viral treatment, synergy was observed. Therefore, timing of administration of oncolytic virus and immunotherapy combinations will need to be refined for progression to clinical trials. More recently, an oncolytic virus encoding for CXCL11, a chemokine known to attract T-cells, was used in combination with an anti-PDL-1 agent against a murine model of peritoneal carcinomatosis (113). The study demonstrated that vvDD-CXCL11 markedly upregulated PDL-1 in the tumor microenvironment due to enhanced T-cell infiltration, and reduced tumor burden when combined with anti-PDL-1. Furthermore, antitumor immunity was observed, with primary tumors growing more slowly in those treated with combination therapy after tumor rechallenge.

### Thermotherapy

The contrast agent, melanin, may facilitate near-IR-assisted thermotherapy in addition to oncolytic virotherapy (88). A near-IR laser was utilized to specifically transfer energy to melanin-induced cells, with the transferred energy consequently converted to thermal energy, eventually heating the melanin-producing cells and cells in their vicinity to temperatures causing protein denaturation and cell death, therefore, enabling thermotherapy. Stritzker et al. demonstrated that aliquots containing cells infected with VACV encoding melanin achieved a higher temperature exposed to laser light, with near-complete kill of all cells within that aliquot as compared to mock-infected cells. They also demonstrated that lung cancer xenografts on tumor-bearing mice treated with the melanin-inducing VACV had significantly enhanced regression when using a single 2-min laser treatment, compared to tumors that were not exposed to the laser light, demonstrating an additive effect.

### Combining VACV with Other Viruses

In another innovative strategy, complementary oncolytic vesicular stomatitis virus (VSV) was combined with oncolytic vaccinia virus to improve therapeutic outcome (70). The two recombinant viral strains synergistically enhanced each other, resulting in better tumor tissue penetration and prolonged survival of tumor-bearing mice. The synergistic effect was, on the one hand, dependent on the VACV B18R gene product which locally antagonizes the innate cellular, antiviral response initiated by type-I IFNs (114–116) and, therefore, supports VSV growth. On the other hand, recombinant expression of the fusion-associated small transmembrane by VSV resulted in enhanced spreading of the VACV. Further studies are needed combining VACVs with other strains of oncolytic viruses to further elucidate potential additive and synergistic treatment effects.

## Vaccinia Viruses in Clinical Trials

Due to the success of vaccinia viruses in preclinical models, there are several ongoing clinical phase I and II studies for human cancer therapy following the treatment with oncolytic vaccinia virus strains including GLONC-1, JX-594, and Pexa-Vex, with promising safety profiles and therapeutic results (Table 2). For example, in a phase I trial using JX-594 in patients with hepatic carcinoma, 3 of 10 patients had a partial response and six had stable disease (117). The primary goals were to determine the maximum-tolerated dose and safety of JX-594 treatment. IT injection of JX-594 into primary or metastatic liver tumors was generally well tolerated, with grade I–III flu-like symptoms reported by all patients, and four patients experiencing transient grade I–III dose-related thrombocytopenia. Grade III hyperbilirubinemia was dose-limiting in both patients at the highest dose. JX-594 replication-dependent dissemination in blood was shown, with resultant infection of non-injected tumor sites. Safety was, therefore, acceptable in the context of JX-594 replication, GM-CSF expression, and systemic dissemination, which led to a phase II trial in patients with unresectable primary hepatocellular carcinoma with promising results (118).

**Table 2:** **Clinical trials with oncolytic vaccinia viruses**.

Condition	Intervention	Phase	Sponsor	Status	Reference
Solid cancers	vvDD-CDSR	Phase I	University of Pittsburgh	Completed	(119)
Hepatocellular carcinoma	JX-594	Phase II	Jennerex Biotherapeutics	Completed	(117, 118)
Metastatic refractory colorectal cancer		Phase I and II			(120)
Refractory solid tumors in pediatric patients		Phase I			(121)
Refractory solid tumors in adults		Phase I			(71)
Malignant melanoma		Phase I and II			(122)

Head and neck cancers	GL-ONC1	Phase I	Genelux Corporation	Completed	(71)
Solid organ cancers with or without Eculizumab		Phase I		Recruiting	(71)
Advanced peritoneal carcinomatosis		Phase I and II		Completed	(71)
Recurrent ovarian cancer		Phase I		Recruiting	(71)
Advanced solid organ cancers		Phase I		Completed	(71)

With Ipilimumab metastatic/advanced solid tumors	Pexa-Vex	Phase I	Centre Leon Berard	Recruiting	(71)
Hepatocellular carcinoma with Sorafenib vs Sorafenib alone		Phase 3			(71)

*Source: www.clinicaltrials.gov*.

## Conclusion

Vaccinia virus has been shown to be a safe and promising anti-cancer agent, facilitating therapy, imaging, and combination treatment, which may help overcome cancer resistance to standard therapy regiments. VACVs’ advantages of a large genomic capacity, fast and efficient replication, and strong safety profile make it an ideal candidate for genetic engineering. Several future generations of oncolytic vaccinia viruses are under investigation, including those armed with immune-stimulating, anti-angiogenic, and prodrug therapy, those encoding reporter genes for the imaging and serial monitoring of oncovirotherapy. Moreover, VACVs are being investigated in combination with various other anti-cancer strategies, including chemo-, radio-, and immunotherapies as well as other oncolytic VACVs. Further study is needed to unlock VACVs’ full potential as part of the future of cancer therapy.

## Author Contributions

The author confirms being the sole contributor of this work and approved it for publication.

## Conflict of Interest Statement

The author declares that the research was conducted in the absence of any commercial or financial relationships that could be construed as a potential conflict of interest.

## Abbreviations

CT, computed tomography; GFP, green fluorescent protein; hNIS, human sodium iodide symporter; hNET, human norepinephrine transporter; hSSTR2, human somatostatin receptor 2; HSV1-tk, herpes simplex virus 1-thymidine kinase; ^124^I, iodine-124; ^125^I, iodine-125; ^131^I, iodine-131; MIBG, meta-iodobenzylguanidine; MRI, magnetic resonance imaging; MSPT, multi-spectral tomography; PET, positron emission tomography; ^99m^Tc_O4_, 99m-technetium pertechnetate; SPECT, single photon emission computed tomography; VACV, vaccinia virus.
